# The Effect of Partial Premixing and Heat Loss on the Reacting Flow Field Prediction of a Swirl Stabilized Gas Turbine Model Combustor

**DOI:** 10.1007/s10494-017-9848-4

**Published:** 2017-09-13

**Authors:** Simon Gövert, Daniel Mira, Jim B. W. Kok, Mariano Vázquez, Guillaume Houzeaux

**Affiliations:** 1 Faculty of Engineering Technology, University of Twente, Enschede, The Netherlands; 2 Computer Applications in Science and Engineering Department, Barcelona Supercomputing Center (BSC), Barcelona, Spain; 3 IIIA-CSIC, Bellaterra, Spain; 4 Present Address: Institute of Propulsion Technology, Combustor, German Aerospace Center (DLR), Cologne, Germany

**Keywords:** Turbulent combustion, Partial premixing, Heat loss effects, Tabulated chemistry

## Abstract

This work addresses the prediction of the reacting flow field in a swirl stabilized gas turbine model combustor using large-eddy simulation. The modeling of the combustion chemistry is based on laminar premixed flamelets and the effect of turbulence-chemistry interaction is considered by a presumed shape probability density function. The prediction capabilities of the presented combustion model for perfectly premixed and partially premixed conditions are demonstrated. The effect of partial premixing for the prediction of the reacting flow field is assessed by comparison of a perfectly premixed and partially premixed simulation. Even though significant mixture fraction fluctuations are observed, only small impact of the non-perfect premixing is found on the flow field and flame dynamics. Subsequently, the effect of heat loss to the walls is assessed assuming perfectly premixing. The adiabatic baseline case is compared to heat loss simulations with adiabatic and non-adiabatic chemistry tabulation. The results highlight the importance of considering the effect of heat loss on the chemical kinetics for an accurate prediction of the flow features. Both heat loss simulations significantly improve the temperature prediction, but the non-adiabatic chemistry tabulation is required to accurately capture the chemical composition in the reacting layers.

## Introduction

In order to meet the stringent regulations related to pollutant emissions while increasing the cycle efficiency, lean premixed combustion is widely used in stationary gas turbine engines. For aeronautical applications, additional space and weight limitations require a maximum efficiency combined with a high compactness of the combustor design. Therefore, a common design approach for flame stabilization is the use of an aerodynamic swirler. The azimuthal momentum introduced by the swirling flow increases the mixing of fuel and oxidizer and creates a low pressure region forming an internal recirculation zone (IRZ) that increases the overall flow residence time. Computational fluid dynamics (CFD) is routinely applied during the development process of new engine designs. However, high fidelity modeling of the turbulence and combustion processes is required to increase the prediction capabilities and reliability for such complex flows. With the increase in computing power, the application of large-eddy simulation (LES) becomes available at the industrial level to model complex combustion systems. LES allows to obtain not only more accurate predictions of mean values, but also evaluate the effects of unsteadiness and fluctuations of important quantities in the flow. However, due to the different scales involved in turbulent combustion problems, the resolution of detailed chemical reactions still remains out of scope for many complex combustion applications. Instead, turbulent combustion models based on flamelet approaches can be used to accurately predict detailed chemical reactions at reduced computational cost [1, 2].

In the current work, a combustion model based on tabulation of laminar premixed flamelets and a presumed-shape Probability Density Function (PDF) to account for subgrid scale (*sgs*) turbulence-chemistry interactions is presented. The model takes chemical kinetics into account by means of an optimized reaction progress variable and an enthalpy variable is utilized to account for heat loss. Mixing is considered in the tabulation using a mixture fraction approach. The model is implemented in a low-Mach number flow solver that is specifically designed for large-scale parallel applications and based on the Finite Element Method (FEM). While the proposed combustion model has already been successfully applied to predict the flow field of a confined premixed jet flame [3], it is now extended to account for non-premixed and partially premixed conditions. While diffusion flames will be addressed in the future, a technically premixed swirl combustor is analyzed in this work as a first validation step. In the technically premixed regime, fuel is injected upstream of the flame and mixes with the air through the swirler before entering the combustion chamber where the mixture ignites. However, even if fuel and air are premixed, the mixture is not necessarily homogeneous and equivalence ratio fluctuations occur.

The test case corresponds to a gas turbine model combustor that has been experimentally investigated at the German Aerospace Center (DLR) by Meier et al. [4]. The combustor has been used extensively as a test case for modeling validation [5–16], while other authors were focused on the description of the flow field and combustion dynamics by means of numerical simulations [17–20]. In the majority of the modeling attempts, perfectly premixing is assumed in order to apply premixed turbulent combustion models. Only few studies have addressed the mixing of fuel and oxidizer with non-premixed combustion modeling. In this line, Franzelli et al. [17] investigated the combustion dynamics for stable and unstable operating conditions using the Dynamically Thickened Flame (DTFLES) model with a reduced chemical scheme. While the fuel-air mixing needs to be considered to capture the thermo-acoustic instabilities, only little impact of the mixing is reported to predict the flow dynamics of the stable flame. Further analysis was carried out by Franzelli et al. [8] with the same setup and combustion model, but with focus on the evaluation of different reduced kinetics. Ansari et al. [6] used this test case in non-premixed conditions for validation of a combustion model based on the Scalar Filtered Mass Density Function (SFMDF) methodology [6]. To the authors knowledge, non-premixed modeling attempts of the current test case using a turbulent combustion model based on tabulated chemistry have not been reported in literature yet. The suitability of this approach for the accurate prediction of the reacting flow field in this configuration is demonstrated by the present investigation. Additionally, not much attention has been yet given to the effect of heat loss on the current test case. Grimm et al. [10] used an isothermal boundary condition in their simulation to account for the heat transfer to the combustor walls obtaining an improvement of the temperature close to the walls. However, the influence of the heat loss on the flame dynamics and chemical kinetics was not further addressed. To the authors knowledge, all other previous investigations are based on the assumption of adiabatic conditions. Due to the neglect of heat loss to the walls, a temperature overprediction is observed in previously reported modeling efforts, especially in the outer recirculation zone (ORZ). Due to long residence times, the effect of heat loss is most significant in this region. Furthermore, with neglected heat transfer to the walls, the temperature fluctuations in the ORZ cannot be predicted accurately.

The analysis presented in this work pursues two major objectives. In the first part, the influence of the fuel-air mixing for this gas turbine model combustor is addressed using large-eddy simulation. The description of the reaction chemistry is based on tabulation of laminar premixed flamelets with presumed-shape PDFs for turbulence-chemistry interaction. The tabulation is coupled to the LES using a mixture fraction and reaction progress variable approach. Thereby, the prediction capabilities of the presented combustion model for partially premixed conditions are demonstrated. In the second part, the influence of heat loss to the walls on the reacting flow field prediction is assessed. For this analysis, a perfectly premixed fuel-air mixture is used as the impact of local mixture fraction inhomogeneities is shown to be very small confirming the results reported in the literature. The heat transfer to the combustor walls is considered by application of isothermal conditions. The results for simulations using non-adiabatic chemistry tabulation are compared to a simulation in which the effect of heat loss on the chemical kinetics is neglected. This allows to analyze the effect of heat loss on the combustion chemistry. In the last step, the impact of radiative heat transfer is discussed by application of a radiation model based on the optically thin gas assumption [21, 22].

The remainder of the paper is organized as follows. The modeling approach is described in Section 2 including the definition of the chemistry controlling variables (Section 2.1), a summary of the governing equations for LES (Section 2.2) and a detailed description of the chemistry tabulation for the different conditions addressed in this work (Section 2.3). Subsequently, the investigated test case is introduced in Section 3 considering the experimental (Section 3.1) and numerical setup (Section 3.2). Afterwards, the results are presented in Section 4 for non-premixed modeling (Section 4.1) and premixed non-adiabatic conditions Section 4.2. Finally, conclusions are given in Section 5.

## Modeling Approach

In the current work, the modeling of the reacting flow field is based on a flamelet approach with tabulated chemistry. Turbulence-chemistry interaction in the *sgs* is accounted for by the use of a presumed-shape PDF. The conservation equations of mass, momentum and enthalpy are solved in the low-Mach number limit and combined with transport equations for the scalar variables controlling the combustion chemistry. In this section, the scalar controlling variables are defined first. Subsequently, the set of governing equations for LES is introduced. Finally, the chemistry tabulation for the different conditions investigated in the current work are presented including the treatment of turbulence-chemistry interaction.

### Controlling variables

The description of the combustion process in the reacting flow is based on the assumption of the flamelet regime and the thermochemical properties of the flame in this region are given from a precomputed flamelet database. The tabulation is parametrized in terms of scalar controlling variables that are used to couple the chemical states with the fluid flow. The controlling variables correspond to the mixture fraction, the reaction progress variable and a normalized enthalpy scalar when accounting for heat loss.

#### Mixture fraction

The mixture fraction describes the local mass ratio between fuel and oxidizer. Based on the element mass fraction *Z*_*j*_, the mixture fraction is defined as:

1 $$ f = \frac{Z_{j}-{Z_{j}^{O}}}{{Z_{j}^{F}}-{Z_{j}^{O}}} $$

where the superscripts *O* and *F* indicate the values at the oxidizer and fuel inlet, respectively. Consequently, the mixture fraction is zero for pure oxidizer and unity in pure fuel flow. In the current work, the assumption of equal diffusivity is utilized. Thereby, the minor effects of differential diffusion are neglected reducing significantly the complexity of the modeling. As demonstrated by Donini et al. [23], the influence of differential diffusion in the modeling of laminar methane flames is negligibly small. In a turbulent flame, the diffusion processes are dominated by turbulent diffusion and the influence of the Lewis number assumption is further reduced. Using the equal diffusivity assumption, any element *j* could be utilized in order to construct the mixture fraction. The mixture fraction definition based on element mass fractions is beneficial because elements are conserved during chemical reaction and, therefore, the mixture fraction does not change due to chemical reactions. This also implies that the mixture fraction is constant along each individual premixed flamelet.

#### Reaction progress variable

The composed species mass fraction *Y*_*c*_, sometimes also referred to as the unscaled progress variable, is defined as a linear combination of species mass fractions *Y*_*k*_:

2 $$ Y_{c} = \sum\limits_{k = 1}^{K} b_{k} Y_{k} $$

where the weighting coefficients *b*_*k*_ describe the contribution of each species *k* to the composed mass fraction *Y*_*c*_ [3]. For premixed conditions, the composed species mass fraction is commonly normalized using the initial and equilibrium conditions. This results in the scaled reaction progress variable *c*:

3 $$ c = \frac{Y_{c} - {Y_{c}^{u}}}{{Y_{c}^{b}} - {Y_{c}^{u}}} $$

where the superscripts *u* and *b* refer to the unburnt and burnt composition, respectively. Using this definition, the reaction progress variable is zero in the limit of unburnt gasses and unity for the fully burnt mixture. For non-premixed conditions, the progress of reaction *Y*_*c*_ depends on the mixture fraction, so the scaling process leads to addition of scalar and cross-scalar dissipation rates in the balance equation of the scaled reaction progress [24]. In order to avoid these additional terms, a transport equation for the unscaled reaction progress *Y*_*c*_ can be solved instead. The latter is the strategy that will be followed here. Details about the transport equations for perfectly premixed and partially premixed conditions are given in Section 2.2.

#### Enthalpy scalar

For non-adiabatic conditions, flamelets at different enthalpy levels are required. The flamelets are parametrized in terms of the normalized enthalpy scalar *i* defined as:

4 $$ i = \frac{h_{min} - h}{h_{min} - h_{max}} $$

and the normalized enthalpy *i* consequently varies from unity for adiabatic conditions (*h* = *h*_*max*_) to zero for maximum heat loss (*h* = *h*_*min*_). Due to the application of the equal diffusivity assumption, the enthalpy is constant along each premixed flamelet. The maximum enthalpy value is determined based on the solution of a freely propagating flame configuration while the minimum enthalpy level for the tabulation is based on a vanishing source term. Consequently, if the enthalpy level is lower than *h*_*min*_, the reaction chemistry remains frozen [3].

### Governing equations

#### Modeling approach for partially premixed flames

The transport equations in the low Mach number regime governing the reactive flow correspond to the conservation of continuity, momentum and enthalpy along with transport equations for the controlling variables describing the mixing and reaction processes. The chemical state is described by the mean and first moment of the mixture fraction and reaction progress respectively. A Favre-filtered description of the governing equations is given to avoid the modeling of terms including density fluctuations [25]. Note that this section describes the governing equations for LES, so a tilde is used for Favre-filtered quantities and an overbar for Reynolds-filtered quantities respectively. In this work, a progress of reaction $\tilde {Y}_{c}$ is preferred over the scale quantity $\tilde {c}$ and by solving the first moment of the progress of reaction $\tilde {{Y_{c}^{2}}}$, the variance of the reaction progress can be obtained as $\tilde {Y_{c}^{\prime \prime 2}}=\tilde {{Y_{c}^{2}}}-\tilde {Y}_{c} \tilde {Y}_{c}$. For the fuel-air mixing, the mixture fraction $\tilde {f}$ and mixture fraction variance $\tilde {f}_{v} = \tilde {f^{\prime \prime 2}}$ are solved to determine the local level of mixing. The modeling approach proposed by Domingo et al. [26] with a linear relaxation hypothesis is used to model the subgrid scale part of the scalar dissipation rate of $\tilde {f}$ and $\tilde {Y}_{c}$ with a filter width expressed in terms of the cell size $\Delta =\sqrt [3]{Vol}$, where *V* *o* *l* is the volume of the cell. No transport equation is required for the enthalpy scalar *i* as it is linked directly to the conservation equation of the enthalpy *h*. Using the assumption of unity Lewis number for all species, the set of filtered balance equations is defined as:

5 $$\begin{array}{@{}rcl@{}} \frac{\partial \bar{\rho}}{\partial t} + {\nabla \cdot} \left( \bar{\rho} \tilde{\boldsymbol{u}} \right) &=& 0 \end{array} $$

6 $$\begin{array}{@{}rcl@{}} \frac{\partial \left( \bar{\rho} \tilde{\boldsymbol{u}}\right)}{\partial t} + {\nabla \cdot}\left( \bar{\rho}\tilde{\boldsymbol{u}}\tilde{\boldsymbol{u}}\right) &=& - \nabla \bar{p} + {\nabla \cdot} \boldsymbol{\tau} + {\nabla \cdot} \boldsymbol{\tau}^{*} \end{array} $$

7 $$\begin{array}{@{}rcl@{}} \frac{\partial \left( \bar{\rho} \tilde{h}\right)}{\partial t} + {\nabla \cdot} \left( \bar{\rho} \tilde{\boldsymbol{u}} \tilde{h} \right) &=& {\nabla \cdot} \left[ \left( \frac{\bar{\lambda}}{\bar{c}_{p}} + \frac{\mu_{t}}{Sc_{t}} \right) \nabla \tilde{h} \right] - \nabla \cdot \boldsymbol{\dot{q}}_{R} \end{array} $$

8 $$\begin{array}{@{}rcl@{}} \frac{\partial \left( \bar{\rho} \tilde{f} \right)}{\partial t} + {\nabla \cdot} \left( \bar{\rho} \tilde{\boldsymbol{u}} \tilde{f} \right) &=& {\nabla \cdot} \left[ \left( \frac{\bar{\lambda}}{\bar{c}_{p}} + \frac{\mu_{t}}{Sc_{t}} \right) \nabla \tilde{f} \right] \end{array} $$

9 $$\begin{array}{@{}rcl@{}} \frac{\partial \left( \bar{\rho} \tilde{Y}_{c} \right)}{\partial t} + {\nabla \cdot} \left( \bar{\rho} \tilde{\boldsymbol{u}} \tilde{Y}_{c} \right) &=& {\nabla \cdot} \left[ \left( \frac{\bar{\lambda}}{\bar{c}_{p}} + \frac{\mu_{t}}{Sc_{t}} \right) \nabla \tilde{Y}_{c} \right] + \tilde{\dot \omega}_{Y_{c}} \end{array} $$

10 $$\begin{array}{@{}rcl@{}} \frac{\partial \left( \bar{\rho} \tilde{f}_{v} \right)}{\partial t} + {\nabla \cdot} \left( \bar{\rho} \tilde{\boldsymbol{u}} \tilde{f}_{v} \right) &= &{\nabla \cdot} \left[ \left( \frac{\bar{\lambda}}{\bar{c}_{p}} + \frac{\mu_{t}}{Sc_{t}} \right) \nabla \tilde{f}_{v} \right] + 2\frac{\mu_{t}}{Sc_{t}} \left|\nabla \tilde{f} \right|^{2} \!- 2 \frac{\mu_{t}}{\Delta^{2} Sc_{t}} \tilde{f_{v}} \end{array} $$

11 $$\begin{array}{@{}rcl@{}} \frac{\partial \left( \bar{\rho} \tilde{{Y^{2}_{c}}} \right)}{\partial t} + {\nabla \cdot} \left( \bar{\rho} \tilde{\boldsymbol{u}} \tilde{{Y^{2}_{c}}} \right) &=& {\nabla \cdot} \left[ \left( \frac{\bar{\lambda}}{\bar{c}_{p}} + \frac{\mu_{t}}{Sc_{t}} \right) \nabla \tilde{{Y^{2}_{c}}} \right] \\ && -2 \left[ \frac{\bar{\lambda}}{\bar{c}_{p}} \left| \nabla \tilde{Y}_{c} \right|^{2} + \frac{\mu_{t}}{Sc_{t} \Delta^{2}} \left( \tilde{{Y^{2}_{c}}} - \tilde{Y}_{c} \tilde{Y}_{c} \right)\right]+ 2 \tilde{Y_{c} { \omega_{Y_{c}}}} \end{array} $$

where $\bar {\rho }$ is the density, $\tilde {\boldsymbol {u}}$ is the velocity vector, $\bar {p}$ is the mechanical pressure, $\bar {\lambda }$ is the thermal conductivity, $\bar {c}_{p}$ is the specific heat capacity, $\boldsymbol {\dot {q}}_{R}$ is the radiative heat flux and $\tilde {\dot \omega }_{Y_{c}}$ is the source term of the reaction progress variable. *S* *c*_*t*_ is the turbulent Schmidt number and a value of 0.7 is used for all simulations presented in this work.

The set of equations is closed by the use of the ideal gas law:

12 $$ \bar{\rho} = \frac{\bar{p}^{th} \bar{W}}{R \tilde{T}} $$

in which *p* ^*th*^ is the thermodynamic pressure, $\bar {W}$ is the mean molecular weight of the mixture, *R* is the universal gas constant and $\tilde {T}$ is the temperature. The temperature is determined by the transport of enthalpy and is evaluated by solving a polynomial expression for the computed enthalpy following:

13 $$ \tilde{h} = \sum \limits_{n = 1}^{5} \frac{a_{n}}{n} \tilde{T}^{n} + a_{6} $$

in which *a*_*n*_ represent the NASA coefficients [27], which depend on the local composition only. Mixture-averaged coefficients are tabulated in the database as a function of the controlling variables. The temperature is computed by inversion of Eq. 13. In practice, an iterative root-finding algorithm based on Newton’s method is used to obtain the temperature from the enthalpy implicitly.

The viscous stress tensor ***τ*** is defined based on Stoke’s assumption and the turbulence contribution ***τ*** ^∗^ is determined by the use of the Boussinesq approximation:

14 $$ \boldsymbol{\tau} = 2 \mu \left[ \boldsymbol{S} - \frac{1}{3} \left( \nabla\tilde{\boldsymbol{u}}\right) \boldsymbol{I} \right] , \quad \boldsymbol{\tau}^{*} = 2 \mu_{t} \left[ \boldsymbol{S} - \frac{1}{3} \left( \nabla\tilde{\boldsymbol{u}}\right) \boldsymbol{I} \right] $$

in which $\boldsymbol {S} = \frac {1}{2} \left [ \nabla \tilde {\boldsymbol {u}} + \left (\nabla \tilde {\boldsymbol {u}} \right )^{T} \right ]$ is the strain tensor and ***I*** is the identity tensor. The turbulent viscosity *μ*_*t*_ is modeled using the Wall-Adapting Local Eddy-viscosity model (WALE) [28].

During the simulation, thermochemical data is extracted from the flamelet database. Next to the filtered reaction source term $\tilde {\omega }_{Y_{c}}$ (or $\tilde {S}_{c}$ for premixed simulations as discussed in the following section), the thermal conductivity $\bar {\lambda }$, the laminar viscosity $\bar {\mu }$, the mean molecular weight $\bar {W}$ and the NASA coefficients *a*_*n*_ are read as function of the controlling variables (Section 2.1). In order to reduce the computational cost during the CFD simulation, mixture averaged values are tabulated directly, while the local species composition is only used for post-processing purposes. The database is generated in a pre-processing step as discussed in the following section.

#### Modeling approach for perfectly premixed flames

In the case of perfectly premixed flames, the four equations controlling the adiabatic combustion process $\phi =\tilde {f}$, $\tilde {f_{v}}$, $\tilde {Y}_{c}$, $\tilde {{Y_{c}^{2}}}$ can be reduced to only two $\phi =\tilde {Y}_{c}$, $\tilde {{Y_{c}^{2}}}$ as no variations of equivalence ratio are assumed in that case. The balance equations for the chemistry can be expressed in terms of the scale reaction progress $\tilde {c}$ and variance $\tilde {c_{v}}$ reading:

15 $$\begin{array}{@{}rcl@{}} \frac{\partial \left( \bar{\rho} \tilde{c} \right)}{\partial t} + {\nabla \cdot} \left( \bar{\rho} \tilde{\boldsymbol{u}} \tilde{c} \right) &=& {\nabla \cdot} \left[ \left( \frac{\bar{\lambda}}{\bar{c}_{p}} + \frac{\mu_{t}}{Sc_{t}} \right) \nabla \tilde{c} \right] + \tilde{S}_{c} \end{array} $$

16 $$\begin{array}{@{}rcl@{}} \frac{\partial \left( \bar{\rho} \tilde{c}_{v} \right)}{\partial t} + {\nabla \cdot} \left( \bar{\rho} \tilde{\boldsymbol{u}} \tilde{c}_{v} \right) &=& {\nabla \cdot} \left[ \left( \frac{\bar{\lambda}}{\bar{c}_{p}} + \frac{\mu_{t}}{Sc_{t}} \right) \nabla \tilde{c}_{v} \right] + \\ && 2 \left( \tilde{S_{c} \, c} -\tilde{S}_{c}\, \tilde{c}\right) + 2\frac{\mu_{t}}{Sc_{t}} \left|\nabla \tilde{c} \right|^{2} - 2 \frac{\mu_{t}}{\Delta^{2} Sc_{t}} \tilde{c_{v}} \end{array} $$

### Chemistry tabulation

The chemistry tabulation is based on the composition of laminar premixed flamelets as proposed by the Flamelet-Generated Manifold (FGM) [2] and Flamelet Prolongation of ILDM (FPI) [1] methods. A low-dimensional manifold is created based on the composition of laminar premixed flamelet structures within the flammability regime, while interpolation between the flammability limits and the inlet values is used outside this range to cover the entire mixture fraction space. This approach allows to address the combustion problem from fully premixed to non-premixed conditions. Only a single flamelet at the global equivalence ratio is required for perfectly premixing fuel-air mixtures (and adiabatic thermal conditions). Contrary, several premixed flamelets are computed to construct the tabulation for non-premixed or partially premixed cases covering the entire range of equivalence ratios within the flammability limits [29]. Similarly, for non-adiabatic conditions, flamelets at different enthalpy levels are tabulated to account for the effect of heat loss on the reaction chemistry [3]. All flamelets are computed by the solution of one-dimensional laminar premixed flame simulations using Chemkin Premix [30] in combination with the GRI-Mech 3.0 reaction mechanism [31].

Subsequently, the chemistry tabulation is discussed. To account for the different conditions that are investigated, three different thermo-chemical databases are used throughout this work. In the following sections, the tabulation process is described with emphasis on the respective conditions.

In the baseline case, the influence of the local mixing is analyzed and compared to the assumption of perfectly premixing under adiabatic conditions. Thereafter, these conditions are relaxed and the influence of heat loss on the reaction chemistry is incorporated for perfectly premixed conditions.

#### Perfectly premixed conditions without heat loss

For perfectly premixed conditions, the mixture fraction is constant and can therefore be omitted. Additionally, using adiabatic thermal conditions, the enthalpy does not vary and the enthalpy scalar can be left out. Therefore, only a single flamelet at the global equivalence ratio is required for the chemistry tabulation using a freely propagating flame configuration. The state of chemical reactions is completely described by the use of a reaction progress variable [2].

In the definition of the reaction progress variable, the weighting coefficients *b*_*k*_ describe the contribution of each species *k* to the composed mass fraction *Y*_*c*_ (see Section 2.1.2). In principle, any linear combination that assures a monotonic behavior of *Y*_*c*_ could be used. In this study, the Computational Singular Perturbation (CSP) mechanism is used to determine the weighting coefficients [32, 33] for perfectly premixed conditions,. Thereby, an optimized choice of the reaction progress variable is obtained that reduces the gradients in the tabulation and lowers the resolution requirements [3]. As a result, almost all species contribute to the definition of the reaction progress variable.

The turbulence-chemistry interaction is based on the application of a presumed-shaped PDF that takes into account the statistical effect of turbulence. For perfectly-premixed adiabatic conditions, the chemistry depends only on the reaction progress variable and an ensemble Favre-filtered scalar is defined as:

17 $$ \tilde{\phi} = \frac{1}{\overline{\rho}}\int \limits_{c = 0}^{1} \rho \phi(c) P(c) dc $$

where the tilde indicates Favre-filtered variables and the overbar denotes Reynolds-filtered quantities. *P*(*c*) is the probability density function of the reaction progress. A *β*-PDF shape is used in the current work to account for the subgrid scale variations of the reaction progress variable, since it was shown that only moderate levels of fluctuations occur for this case [34]. The *β*-PDF is defined by the filtered reaction progress variable $\tilde {c}$ and the *sgs* variance of the reaction progress variable $\tilde {c}_{v}$. The dimension of the resulting turbulent database is therefore increased. For the Favre-filtered reaction progress variable and its variance, transport equations are formulated as presented in Section 2.2. The Favre-filtered chemical source term is the presumed-shape PDF weighted chemical source term computed from the corresponding laminar flame solution. To solve for the local temperature and enthalpy, the coefficients *a*_*n*_ of Eq. 13 are read from the laminar database as a function of the Favre-filtered progress variable.

In the turbulent database, the chemistry is tabulated at 100 discrete points in reaction progress space with a linear subdivision. For each of these points, the variance of the reaction progress variable is tabulated at 25 discrete values. Since the *β*-function differs greatly in shape for small values of the variance, a cubic subdivision of the grid points is chosen for the variance distributing most of the points between zero and 0.25 [34].

**Fig. 1:**
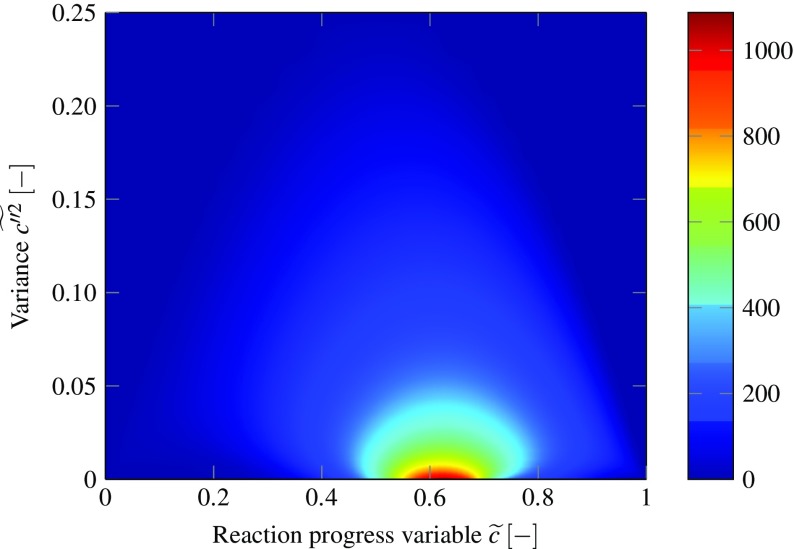
Source term of reaction progress variable $\tilde {S}_{c} \; \left [\frac {\text {kg}}{\mathrm {m}^{3} \mathrm {s}}\right ]$ as function of mean and variance of reaction progress $\tilde {c}$

The turbulent database for perfectly premixed, adiabatic conditions is visualized in Fig. 1 on basis of the source term of the reaction progress variable. The laminar conditions correspond to a variance of zero, while an increased value of the variance can be understood as an increased turbulence level. With increased variance values, the filtered maximum source term value decreases due to the convolution with the PDF. Additionally, the source term is distributed over a wider range due to a smearing effect that reduces the source term gradient and provides increased numerical stability and less stiffness.

#### Non-premixed conditions without heat loss

**Fig. 2:**
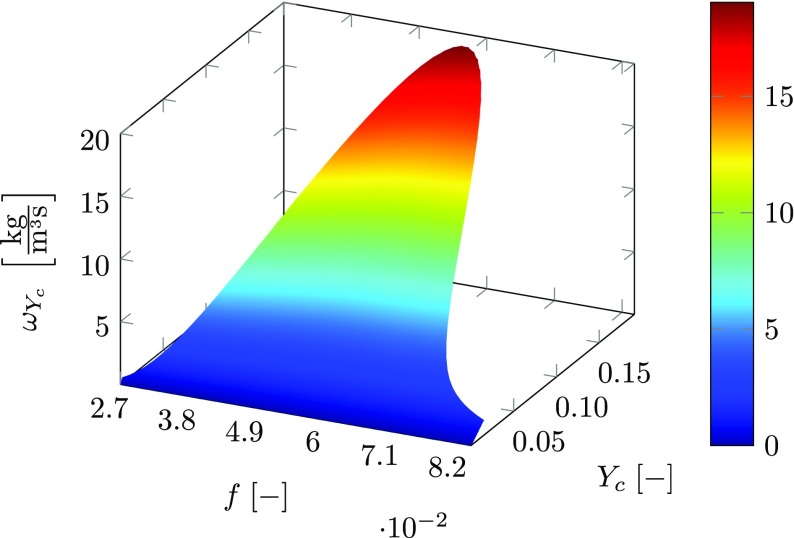
Source term of unscaled reaction progress variable $\omega _{Y_{c}} \; \left [\frac {\text {kg}}{\text {m}^{3}\,\text {s}}\right ]$ as function of mixture fraction *f* and unscaled progress variable *Y*_*c*_ for non-premixed, adiabatic conditions

To take into account the effect of non-perfect mixing of fuel and oxidizer, premixed flamelets at different equivalence ratio are computed and assembled to cover the entire range of equivalence ratio within the flammability limits. The degree of mixing is parametrized in terms of the mixture fraction *f* as introduced in Section 2.1.1. For the non-premixed tabulation, the CSP method is not easily applied due to the different dominant reactions and species for lean and rich conditions and the resulting differences in composition. Therefore, a manual definition of the reaction progress variable is employed to ensure a monotonic behavior of the flamelets across the entire flammability range. The definition employed for the current case reads:

18 $$ \eta = \frac{Y_{H_{2}}}{W_{H_{2}}} + \frac{Y_{H_{2}O}}{W_{H_{2}O}} + \frac{Y_{CO_{2}}}{W_{CO_{2}}} $$

where *W*_*k*_ is the molecular weight of species *k*. The effect of non-perfect premixing can be assessed based on the source term of the reaction progress variable presented in Fig. 2. It spans over the mixture fraction range from *f* = 0.027 up to 0.082. Around the stoichiometric mixture fraction, *f*_*st*_ = 0.055 for the current case, the highest values of the source term are obtained and steep source term gradients indicate small chemical time scales. Contrary, in the limits of the tabulated mixture fraction, the source term tends toward zero, corresponding to the lean and rich flammability limits. Outside the flammability range, the reaction source term is zero and only mixing is considered. This is achieved by linear interpolation of the properties tabulated for the lean or rich limit and the inlet properties of the oxidizer or fuel inlet, respectively. Due to this treatment, a significant reduction of the entries in the database is obtained.

For non-premixed calculations, the chemical evolution represented by the laminar database depends on the the mixture fraction *f* and the scaled reaction progress variable *c*. Therefore, the PDF formulation to account for turbulence-chemistry interaction is defined by the joint PDF *P*(*f*,*c*). Commonly, *f* and *c* are considered statistically independent [35, 36], which allows to split the joint PDF into a factorized PDF *P*(*f*)*P*(*c*). The presumed *β*-PDF is then applied separately for both one-dimensional PDFs. Transport equations are solved for the filtered progress of reaction $\tilde {Y}_{c}$ and its first moment $\tilde {{Y_{c}^{2}}}$ and mixture fraction $\tilde {f}$ and its variance $\tilde {f}_{v}$ as described in Section 2.2. In order to access the database, the scale quantities are computed as:

19 $$ \tilde{c} = \frac{\tilde{Y}_{c}}{Y_{c}^{eq}} \; , \qquad \tilde{c}_{v} =\frac{\tilde{{Y^{2}_{c}}}}{(Y_{c}^{eq})^{2}} - \frac{\tilde{Y}_{c}}{Y_{c}^{eq}} \frac{\tilde{Y}_{c}}{Y_{c}^{eq}} \; , \qquad $$

The integrated variables are stored in a turbulent database at 25 discrete points for $\tilde {f}_{v}$ and $\tilde {c}_{v}$ with a cubic subdivision between zero and 0.25. A number of 58 discrete values are used for $\tilde {f}$ in-between the flammability limits with a linear subdivision, while 130 discrete points are chosen for the tabulation of $\tilde {c}$. To capture the steep gradients of the source term for high values of the reaction progress, about 80% of the points are placed in the region above $\tilde {c} = 0.5$.

#### Perfectly premixed conditions including heat loss

For non-adiabatic conditions, several flamelets at different enthalpy levels are determined by the solution of burner stabilized one-dimensional laminar premixed flame simulations with varying mass flow rate [3, 37, 38]. The flamelets are parametrized in terms of the normalized enthalpy scalar *i* as defined in Section 2.1.3.

**Fig. 3:**
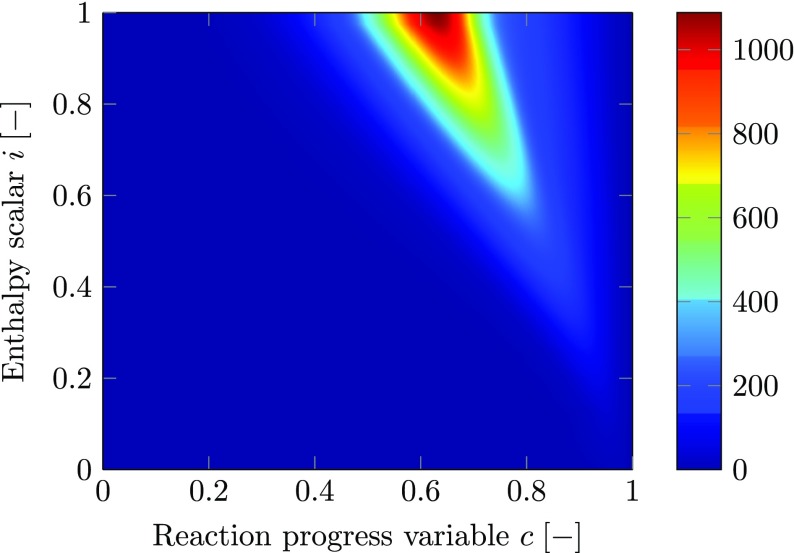
Source term of reaction progress variable $S_{c} \; \left [\frac {\text {kg}}{\text {m}^{3}\, \text {s}}\right ]$ for perfectly premixed, non-adiabatic conditions

In Fig. 3, the perfectly premixed, non-adiabatic database is visualized based on the source term of the reaction progress variable. It is plotted as function of the enthalpy scalar and the reaction progress variable allowing an analysis of the heat loss effect on the chemical kinetics. With increased heat loss (reduced value of the enthalpy scalar) the absolute value of the source term is reduced meaning that the overall chemical reaction rate reduces. Additionally, the peak value of the source term is shifted to higher values of the reaction progress variable.

Statistically independent fields are again assumed in order to apply a factorized PDF for reaction progress variable and enthalpy scalar. In general, only weak dependency is expected for enthalpy and reaction progress as the enthalpy is only changing due to heat losses, which are independent of the reaction progress. Furthermore, heat loss occurs mainly in regions in which the reaction progress already equals unity and the reaction progress fluctuations are zero. Additionally, statistical correlations caused by the geometry of the domain or physically allowed values are eliminated by the normalization of the variables. Due to the almost linear dependency of the species mass fractions and temperature on the enthalpy scalar *i*, turbulent fluctuations in *i* have only a small effect [39] and a *δ*-PDF is employed for the enthalpy. The main advantage of using the *δ*-PDF is that it only depends on the mean of the enthalpy scalar and higher moments do not need to be computed. The turbulent database is stored at 100 discrete points for $\tilde {c}$, 25 points for $\tilde {c}_{v}$ and at 40 discrete enthalpy levels between *h*_*min*_ and *h*_*max*_ using a linear subdivision.

### Numerical approach

The system of equations described in the previous sections is solved using the multiphysics code Alya. The space discretization is based on the variational multiscale method (VMS) using linear finite elements along with the WALE model [28] to take into account subgrid scale effects. The time discretization is based on a second order Backward Finite Difference (BFD) scheme. The parallelization strategy follows a Master-Worker interaction model [40]. In this study, a full MPI strategy is employed, where each MPI task (or Worker) is in charge of each subdomain. The Workers build the local matrices (A_*i*_) and right-hand side (b_*i*_), and control the resulting system solution in parallel. Details of the parallel performance and computational efficiency of the code can be found in [41].

## Test Case Description

The current work is focused on the analysis of the flow field and the influence of heat loss and fuel-air mixing for the PRECCINSTA burner, which is a gas turbine model combustor derived from an industrial design by Turbomeca [42, 43]. The next subsections describe the experimental configuration and the corresponding numerical setup.

### Experimental setup

**Fig. 4:**
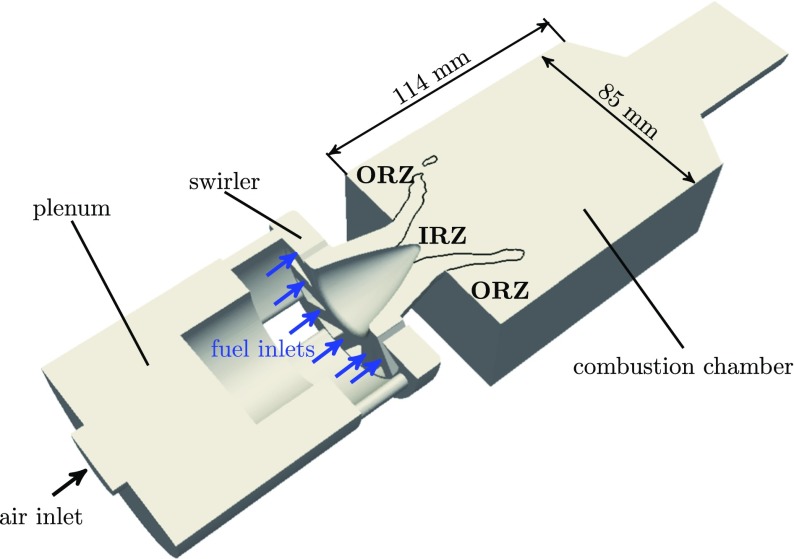
Sketch of one half of the burner as used in the numerical simulations. It includes plenum, swirler and combustion chamber with the main dimensions. The air and fuel inlets are visualized by arrows. The flame location is indicated and the inner and outer recirculation zones are marked

The gas turbine model combustor investigated in this paper has been experimentally investigated at the German Aerospace Center (DLR) by Meier et al. [4]. The combustor is operated at atmospheric pressure and consists of a plenum, a radial swirler, a square combustion chamber with a cylindrical outlet. A sketch of the combustor is presented in Fig. 4. The combustion chamber has a square cross section of 85 × 85 mm and a length of 114 mm. The chamber walls are made of synthetic quartz glass to allow optical access for laser-based measurements. Air at ambient temperature is fed to the burner nozzle through the plenum and swirler. Methane is used as fuel gas and it is injected with high momentum in each of the 12 radial swirler vanes through small holes with a diameter of 1 mm. Fuel and air are mixed up in the swirler vanes before entering the combustion chamber and only small mixture fraction variations are observed in the measurements at this operating point.

The azimuthal momentum introduced by the radial swirler forms an inner and outer recirculation zone. Thereby, the flame is stabilized and anchored at the central bluff body of the burner nozzle. The burner is operated at globally lean equivalence ratio. Depending on the fuel-to-air ratio, different combustion dynamics are observed. For a range of operating conditions, the burner is running stable with a quiet flame. However, strong self-excited thermoacoustic oscillations occur for certain conditions. Both regimes have been investigated in the experiments, but we focus on the stable flame only, as model validation and evaluation of the effects of heat loss and fuel-air mixing is mainly pursued in this study. The measurements of the quiet flame were performed for two operating conditions with slightly different equivalence ratios. The laser Raman scattering measurements of major species concentrations and temperature were performed for *ϕ* = 0.83, while the three dimensional velocity field was measured at *ϕ* = 0.75 using Laser Doppler Velocimetry (LDV). The air mass flow rate was kept constant for both equivalence ratios and the corresponding flame parameters are summarized in Table 1.

**Table 1:** Flame parameters

Flame	Air mass flow	CH_4_ mass flow	*P*_*th*_	Φ_*glob*_	*f*_*glob*_	*T* *g* *l* *o* *b* *a* *d*
	[g/min]	[g/min]	[kW]	[–]	[–]	[K]
A	734.2	35.9	30.0	0.83	0.0463	2037
B	734.2	32.3	27.0	0.75	0.0418	1915

The temperature and major species are measured in vertical planes at eight different heights downstream of the injector (*h* = 6, 10, 15, 20, 30, 40, 60 and 80 mm). In contrast, the LDV measurements of the velocity field are conducted at six different axial locations (*h* = 1.5, 2.5, 5, 15, 25, and 35 mm), which differ from the locations of the Raman measurements. The systematic and statistical measurement uncertainties are below 4% and 2.5% for the temperature, less than 5% and 7% for major species (with the exception of CO and H_2_ for which the systematic and statistical uncertainties can reach values of 10% and 50%, respectively) and not more than 0.5% and 2% for the velocity measurements, respectively [4].

### Numerical setup

The numerical domain is derived from the experimental test rig and presented in Fig. 4. It includes the plenum, the swirler and the combustion chamber. Infeasibly small mesh elements would be required to accurately resolve the flow in the narrow fuel inlet pipes. Therefore, the fuel inlet pipes are not included in the computational domain, but the fuel is directly injected using boundary patches on the swirler vanes.

**Fig. 5:**
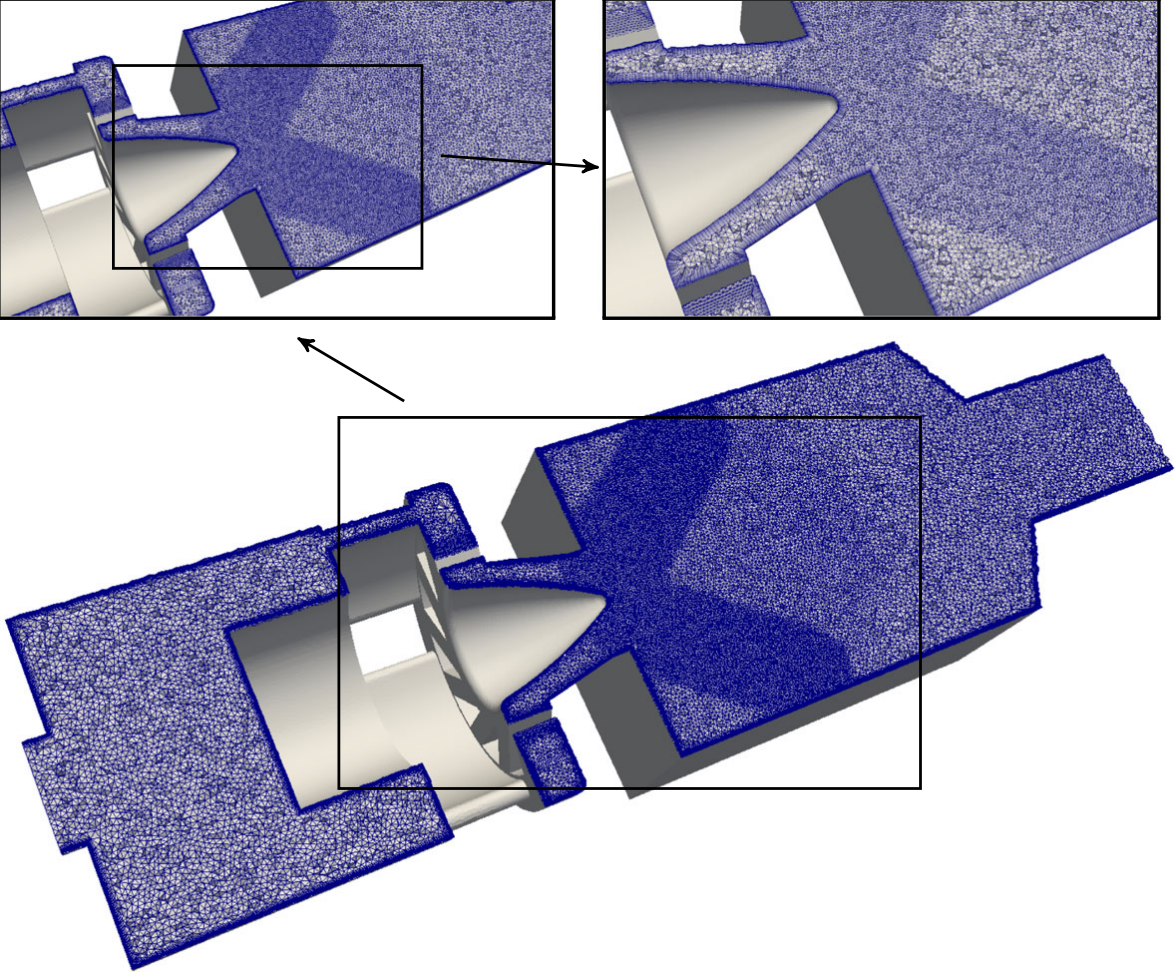
Cut through the computational domain showing the computational mesh for the premixed case with close up views of the different refinement regions in the combustion chamber and swirler section

The computational domain is meshed with tetrahedral elements in the bulk volume. Layers of prism elements at the walls are added in order to resolve the boundary layer. This is especially important to correctly predict the flow separation at the cone of the inlet nozzle, which has significant impact on the flame spreading and the expansion of the inner recirculation zone. The mesh for the premixed simulations contains a total number of 16 million (M) elements and is presented in Fig. 5. It is the result of a mesh dependency analysis in which several meshes at different refinement levels were tested. In the combustion chamber, small elements are placed in the flame region, which gradually coarsen further downstream. A different mesh is used for the non-premixed simulations, in which the fuel inlet patch is added. It is further refined at the fuel injection location and in the swirler vanes to capture the mixing of fuel and air. The non-premixed simulation is run on a refined mesh in which the element size in the flame and mixing region is reduced to 0.45 mm resulting in a total of 29M elements. As small deviations in mixture fraction have large implications on the combustion chemistry (see Fig. 2 in Section 2.3.2), higher mesh resolution is required for a correct representation of the fuel-air mixing as compared to the perfectly premixed conditions.

The inlet velocity is specified with a top hat profile at the fuel and air inlet such that the mass flow rates specified in Table 1 are satisfied. For the perfectly premixed simulations, the entire mass flow is injected at the air inlet. Even though the air is injected at ambient temperature in the experiments, the mixture is slightly preheated by the contact with hot combustor components before entering the combustion chamber [4]. The inlet temperature for the simulations therefore slightly increases and a value of 320 K for fuel and air stream is used as proposed by Franzelli et al. [17]. No slip boundary conditions are specified at the walls for all velocity components. Adiabatic thermal conditions are applied for the simulations in which heat loss is not considered. For the non-adiabatic simulation, an isothermal condition is used for the outer quartz glass walls. The exact wall temperature was not measured in the experimental study and is therefore unknown. However, in previous studies, only limited influence of the exact wall temperature in the combustion chamber was found [44]. Therefore, a wall temperature of *T*_*w*_ = 1000 K is prescribed for all quartz glass walls of the combustion chamber for the isothermal cases.

## Results

In this section the simulation results of the radial swirl burner are presented. In the first part, the influence of non-perfect mixing on the reacting flow field is examined using the modeling approach for partially premixed flames. The second part is focused on the effect of heat loss on the dynamics and chemical structure of the flame.

### Partial premixing

In the experiments, fuel is injected into the swirler vanes and mixes with the air before the mixture enters into the combustion chamber. However, the fuel-air mixture is not homogeneous yet and the combustor is considered to operate under technically premixed conditions. In this section, the influence of the fuel-air mixing on the reacting flow field is assessed and the prediction capabilities of the presented combustion model are demonstrated. Therefore, simulations based on the assumption of perfectly premixing are compared to partially premixed simulations, in which the fuel-air mixing is solved by governing equations. The experimental data serves as a reference.

**Fig. 6:**
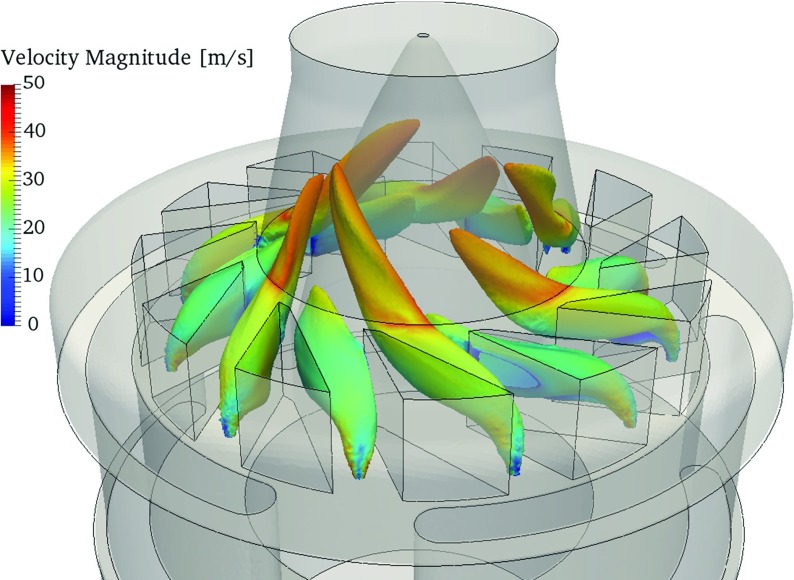
Isosurface of stoichiometric mixture fraction *f*_*st*_ = 0.055 colored by the time averaged velocity magnitude for *ϕ* = 0.83

For the perfectly premixed simulations, a homogeneous fuel-air mixture at the global equivalence ratio is injected directly into the combustion chamber. Contrary, for the technically premixed case, the fuel is injected into the air in the swirler vanes. Figure 6 illustrates the mixing of fuel and oxidizer based on the isosurface of mean stoichiometric mixture fraction *f*_*st*_ = 0.055. The color scale corresponds to the velocity magnitude. The fuel injection in the swirler vanes is arranged as a jet-in-crossflow configuration. Therefore, small stagnation regions indicated by low velocity develop upstream of the fuel jet. The azimuthal momentum induced by the swirler results in a helicoidal motion of the fuel jets that enhance the mixing with air. Thereby, an overall lean mixture is obtained already before entering the combustion chamber.

**Fig. 7:**
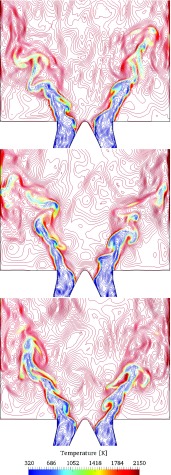
Instantaneous iso-contours of axial velocity colored by temperature. The different time instances are *t* = 0.0868 s (top), *t* = 0.0992 s (middle) and *t* = 0.1116 s (bottom). The results correspond to the partially premixed conditions with *ϕ* = 0.83

In Fig. 7, the instantaneous iso-contours of the axial velocity at different time instances are presented. The contours are colored by temperature to indicate the flame location. The flame is anchored at the central bluff body of the inlet nozzle. The flame surface is wrinkled by the large scale eddies formed in the shear layers of the inner and outer recirculation zones. In agreement with the observations reported by Steinberg et al. [45] and Oberleithner et al. [46], no precessing vortex core (PVC) is observed for the current operating conditions. Instead, the dominant dynamics of flow structure is the vortex shedding in the shear layers. Clearly observed is the anchoring of the flame at the central bluff body.

The comparison of the experimentally measured quantities such as velocity, temperature, mixture fraction or species is based on two different operating conditions. This is because LDV and Raman measurements have been performed for slightly different equivalence ratios (see Table 1 in Section 3.1). Therefore, the comparison of the velocity field is based on the global equivalence ratio *ϕ* = 0.75 (Flame B) while the assessment of temperature and major species prediction is carried out for a global equivalence ratio of *ϕ* = 0.83 (Flame A). Based on these two operating conditions, the results of the perfectly premixed and partially premixed simulations are compared next.

**Fig. 8:**
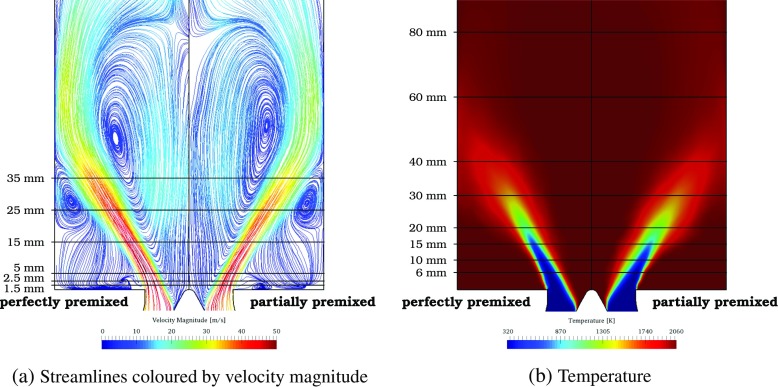
Comparison of time-averaged fields for perfectly premixed and partially premixed simulation. Dashed lines indicate the measurement stations for comparison with the experimental data

In Fig. 8, the time-averaged solution fields of velocity (based on streamlines colored by the velocity magnitude) and temperature are presented and compared for the perfectly and partially premixed conditions. To avoid any influence of small asymmetries, the same half of the combustor is presented and the results are mirrored. The velocity streamlines indicate three distinctive flow regions in the combustion chamber. The azimuthal momentum induced by the swirler vanes results in a conically shaped inlet flow characterized by high velocities. Thereby, an inner recirculation zone (IRZ) is formed in the center of the combustion chamber and outer recirculation zones (ORZ) at the corners. Hot combustion products are transported upstream of the recirculation zones stabilizing the flame.

The comparison of the perfectly premixed and partially premixed simulation indicates very similar solution fields and no distinctive qualitative differences can be observed as demonstrated by Franzelli et al. [17]. For a quantitative comparison, the profiles at different heights are compared to the experimental reference data. The exact location of the profiles are indicated by the dashed lines in Fig. 8.

**Fig. 9:**
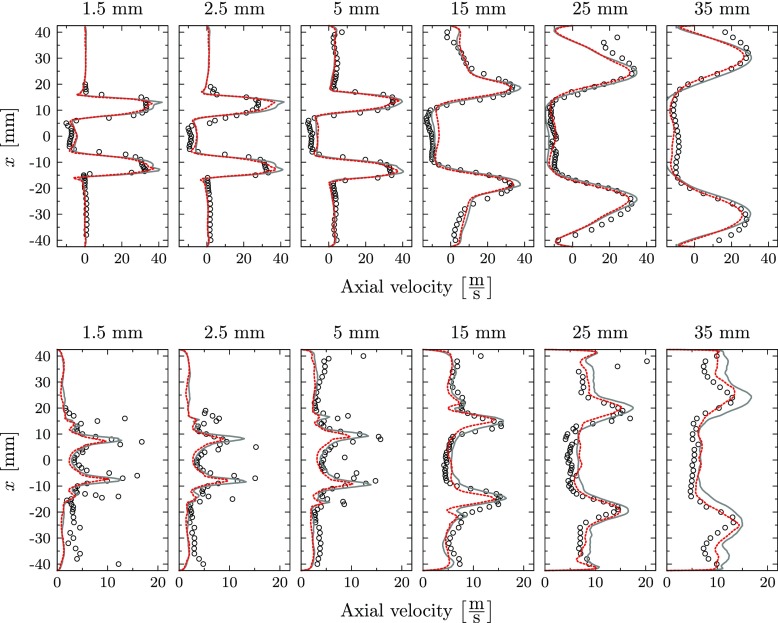
Mean axial velocity (top) and RMS profiles (bottom): Experiments (), perfectly premixed simulation () and partially premixed simulation ()

**Fig. 10:**
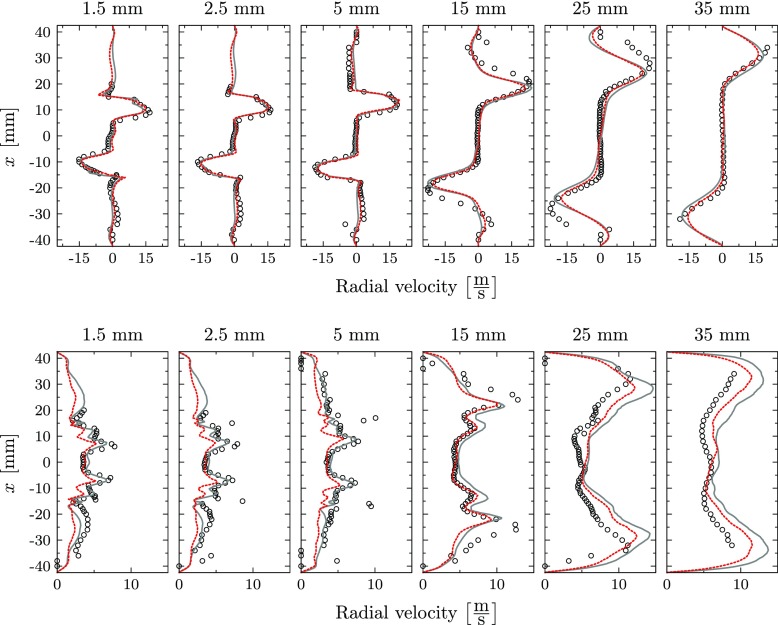
Mean radial velocity (top) and RMS profiles (bottom): Experiments (), perfectly premixed simulation () and partially premixed simulation ()

**Fig. 11:**
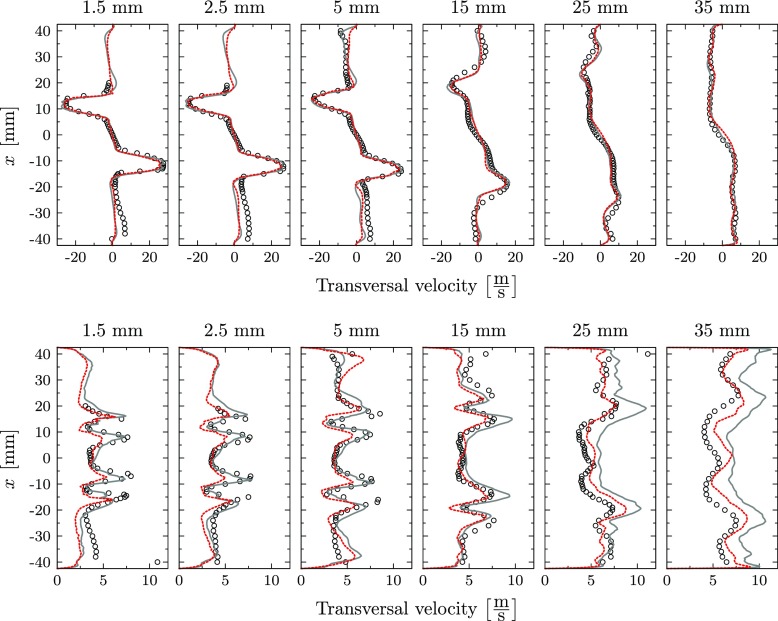
Mean transversal velocity (top) and RMS profiles (bottom): Experiments (), perfectly premixed simulation () and partially premixed simulation ()

The comparison of the axial, radial and transversal velocity components with the experiments is presented in Figs. 9, 10 and 11 for mean and root mean square (RMS) values. The predicted mean fields reveal good agreement with the measurements for all velocity components. The location and strength of all peak values are accurately captured by the perfectly and partially premixed simulation and only small differences between the simulations are observed. Also the correspondent RMS profiles are in good agreement with the reference LDV data, indicating that the same underlying fluid dynamics are predicted by the simulations. However, close to the nozzle exit, the strength of the RMS peak related to the location of the outer reacting layer is underpredicted for the axial velocity by both simulations. In general, the highest deviations on the RMS values are observed at locations with strong temperature intermittency. The same observation was obtained by Roux et al. [20], who related this discrepancy to the Favre filtering of the LES variables.

At all locations, the partially premixed simulation predicts slightly lower RMS peak values compared to the perfectly premixed case for all velocity components. Compared to the experiments, the RMS magnitude is more accurately predicted by the perfectly premixed simulation for the locations close to the jet exit. At these locations, the RMS magnitude is slightly underpredicted in the partially premixed simulation. On the other hand, the partially premixed simulation is closer to the experiments for downstream locations in which the RMS values are slightly overpredicted by the perfectly premixed simulation.

**Fig. 12:**
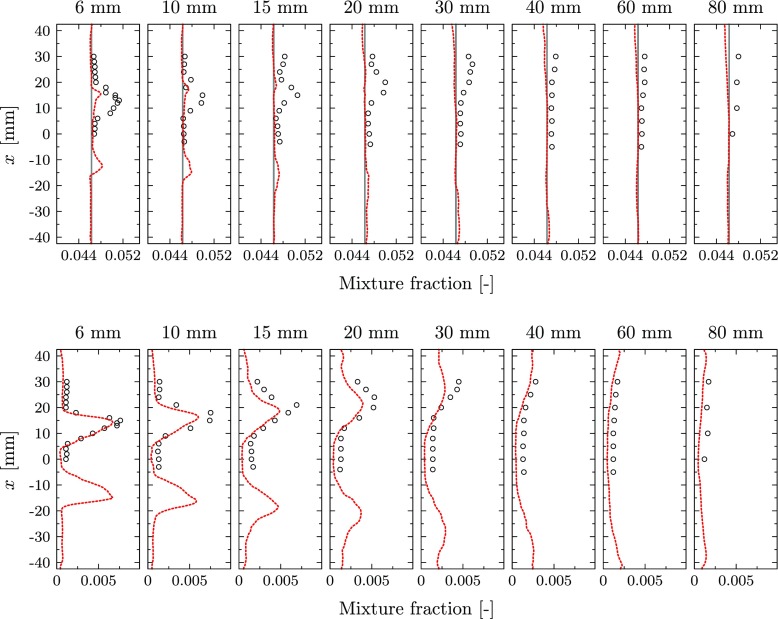
Mean mixture fraction (top) and RMS profiles (bottom): Experiments (), perfectly premixed baseline case () and partially premixed simulation (). Note that the mixture fraction is not explicitly used in the perfectly premixed simulation and the respective profile is only added as a reference

**Fig. 13:**
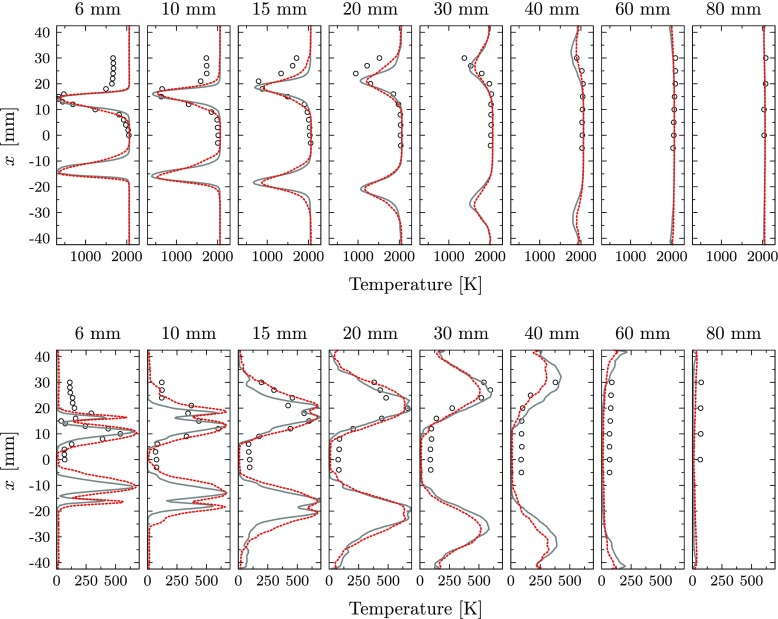
Mean temperature (top) and RMS profiles (bottom): Experiments (), perfectly premixed () and partially premixed simulation ()

The comparison of the mixture fraction and the temperature with the experimental data is presented in Figs. 12 and 13. Note that the results are for a different operating point compared to the velocity plots. The mixture fraction is only used for the partially premixed simulation, while a constant and uniform value is prescribed in the perfectly premixed case. However, the global mixture fraction is added to the mean profiles presented in Fig. 12 to serve as a reference. The profiles of mean mixture fraction reveal an almost constant value in the entire combustion chamber for both, simulation and experiments. Only close to the inlet nozzle, a small mixture fraction peak is observed for the experiments. The strength of the peak is underpredicted by the simulation. The mean mixture fraction predicted by the simulation is in general slightly lower compared to the experiments. However, the simulation results match quite accurately the global value that is added as reference, while the measured values are slightly higher at all locations.

In general, the mixture fraction fluctuations are accurately captured by the simulation and the peak locations of the mixture fraction RMS are correctly predicted. Only the peak values in the reacting layers are slightly underpredicted by the simulation. This mismatch is highly influenced by the mixing process in the cross-flow configuration at the swirler vanes that requires extremely fine resolutions to capture the exact velocity profiles and turbulent intensity responsible for the mixing process. The measured RMS takes a maximum value of about 16% of the mean value, indicating relatively strong mixture fraction fluctuations at the inlet of the combustion chamber. A small underprediction of the peak value and the inner recirculation zone is observed for the simulation, but the general unsteady mixing behavior is captured accurately.

The predicted mean temperature is also in good agreement with the experimental data for the perfectly premixed and partially premixed simulation. The results indicate a correct prediction of the flame shape and location. Only in the ORZ for both simulations overpredict the temperature due to the neglected heat loss. The equilibrium temperature is correctly predicted by the simulations and it is in alignment with the measurements. The peak values and its locations for the RMS temperature are accurately captured for both simulations. The differences between the two cases are small, but similar to the velocity fluctuations. While the perfectly premixed case predicts slightly higher RMS peak values in the downstream profiles, slightly larger flame spreading is formed in the upstream profiles for the partially premixed simulation, leading to improved correlation with the experimental reference. Hardly any fluctuations are predicted in the ORZ due to the neglected heat loss to the walls.

**Fig. 14:**
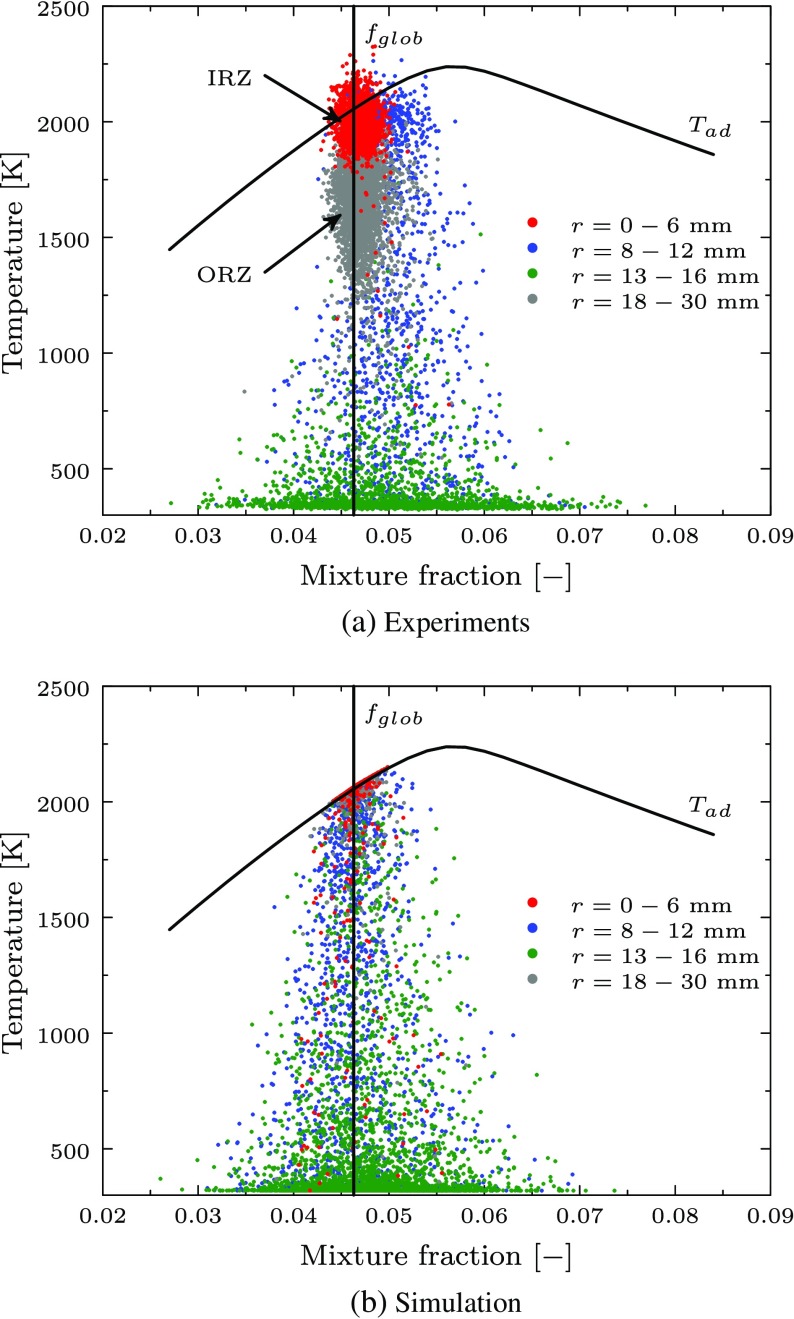
Scatter plot at *h* = 6 mm above the burner exit. The global mixture fraction and the adiabatic flame temperature are marked. The IRZ and ORZ are indicated in the scatter plot based on the experimental data

In Fig. 14, the scatter plots at the horizontal plane 6 mm above the burner nozzle are compared for experiments and partially premixed simulation. Separate colors are used to roughly identify the different regions: IRZ, mixing zone, reacting layers and ORZ. The global equivalence ratio *f*_*glob*_ = 0.0463 is indicated by the vertical line and the adiabatic flame temperature is determined based on equilibrium computations using Chemkin Equil [30]. It is observed that the points corresponding to the mixing and shear layer regions are shifted slightly to the lean side of the global equivalence ratio. This corresponds to the small deviation in Fig. 12, where the small peak in mixture fraction for the range 6 < *x* < 18 mm is not predicted by the simulation. However, the maximum mixture fraction spreading observed in the experiments and the simulation is almost identical with values between 0.03 and 0.075. Also the temperature spreading in this region is comparable for experiments and simulation and characterizes the intermediate state of reaction. The mixture fraction for the points in the IRZ and ORZ are scattered around *f*_*glob*_ for simulation and experiments. While the temperature in the IRZ is at the adiabatic equilibrium in the simulation and the experiments, the temperature for the ORZ is scattered around 1500 K in the experiments. Besides, the temperature in the simulations is very close to the adiabatic flame temperature for most points in the ORZ. The higher temperature predicted by the simulation is caused by the neglected heat transfer to the surroundings, which is most significant in the ORZ due to high residence times close to the walls. In the experiments, temperatures above the adiabatic flame temperature are observed. This can be explained by local heat transfer between the gas mixture and different preheating due to the contact with hot combustor components. Both effects are not accounted for in the numerical model and in the simulation the maximum temperatures can not exceed the adiabatic equilibrium.

**Fig. 15:**
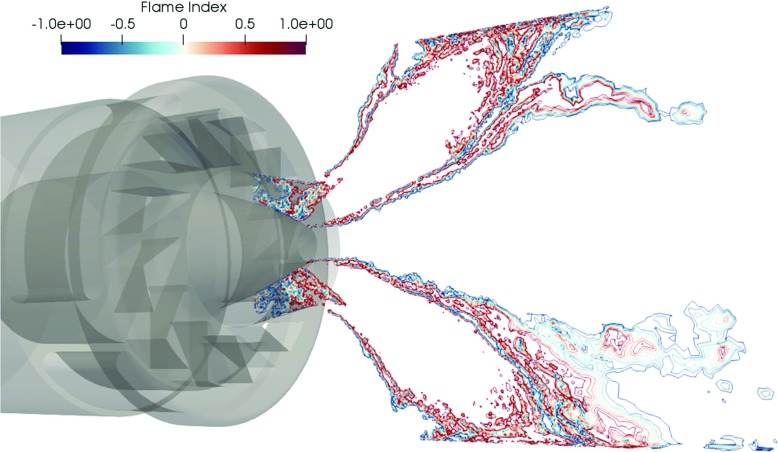
Flame index computed from *Y*_*f*_ and *Y*_*O*_ using the partially premixed turbulent combustion model

A significant scattering of the mixture fraction is observed in Fig. 14 with variations between 0.03 and 0.075. Additionally, the maximum RMS values in Fig. 12 are about 16% of the mean mixture fraction. This demonstrates that the mixing in the combustor is not perfect and contains inhomogeneities in the fuel-air mixture. Nevertheless, the prediction of the velocity field and the temperature is almost identical using the assumption of perfectly premixed conditions as compared to the partially premixed simulation. This is in agreement with the observations by Franzelli et al. [17] who reported a significant influence of the fuel-air mixing only for unstable operating conditions. Also in the experimental study of Steinberg et al. [45] only small differences are found in the measured flow field when a perfectly premixed mixture was injected compared to the technically premixed configuration. In order to further evaluate the contribution of the premixed combustion compared to the diffusion burning on this flame, the flame index from Yamashita et al. [47] is scaled and presented in Fig. 15. This index is computed as:

20 $$ FI = \frac{\nabla Y_{f} \cdot \nabla Y_{O}}{\rvert\nabla Y_{f} \cdot \nabla Y_{O}\rvert} $$

where *Y*_*f*_ and *Y*_*O*_ are the fuel and oxidizer mass fractions, respectively. The flame index shows positive values for regions dominated by flame propagation, and negative values for diffusion dominated flames. The plots show the distribution of combustion regimes over the reacting layer. It is observed that the premixed combustion dominates the reacting process because fuel and oxidizer are aligned, and only in the post flame region, the flames are influenced by diffusion and strain. The results in Figs. 9, 10, 11, 12 and 13 indicate the local equivalence ratio variations have a small influence on this case.

### Heat loss effects

In the previous section, only small impact of the fuel-air mixing on the prediction of the reacting flow field is observed and the condition for perfectly premixed burning holds for this operating point. However, the influence of heat loss is fundamental for this combustor. It was shown that adiabatic thermal conditions lead to an overprediction of the mean temperature close to the walls, which is most significant in the ORZ. Additionally, the temperature fluctuations are not captured in this region as indicated by the low RMS values. In this section, the effect of heat loss for the current test case is addressed. As the impact of fuel-air mixing on the reacting flow field is small, a perfectly premixed mixture is assumed in this section. Thereby, the computational and modeling requirements are substantially reduced. The heat loss to the quartz glass walls is taken into account by application of isothermal wall boundary conditions as described in Section 3.2. The analysis is based only on the operating point with equivalence ratio *ϕ* = 0.83 for which the Raman measurements have been performed. Only little sensitivity of small changes in the operating conditions was observed for the velocity fields, hence the current analysis will be focused on the comparison with the Raman data.

The impact of heat loss on the reacting flow field prediction is assessed by the comparison of the cases summarized in Table 2. In the implementation of the combustion model, a transport equation for the enthalpy is combined with a temperature computation based on mixture averaged NASA polynomials (refer to Section 2.2 for more details). Thereby, heat loss to the environment can be accounted for in different ways. If the impact of heat loss on the flame is small, the adiabatic chemistry tabulation can be used. In this case, the temperature field is driven by the heat loss to the walls, but the interaction between heat loss and chemical kinetics is neglected (Case 2). The comparison of this approach with a simulation in which the effect of heat loss on the reaction chemistry is additionally considered (Case 3) allows to assess the implication of heat loss on the kinetics and the chemical structure of the flame for the investigated gas turbine model combustor. Additionally, the influence of radiative heat transfer on the temperature prediction in the combustor is addressed by the use of a radiation model based on the optically thin flame assumption (Case 4). The optically thin flame model is based on the addition of a volume source term in the enthalpy transport equation describing the divergence of the radiative heat flux [21, 22]. A detailed description of the applied model can be found in [44]. The computation of the absorption coefficient is based on the polynomial functions provided by Chen et al. [48]. CO_2_ and H_2_O are considered as the main radiative participant species and the influence of other species is neglected.

**Table 2:** Heat loss cases

Case	Description
1	Baseline case assuming adiabatic thermal conditions
2	Isothermal wall boundary conditions but neglecting the effect of heat loss on the chemical kinetics
3	Isothermal wall boundary conditions with non-adiabatic chemistry tabulation as described in Section 2.3.3
4	Isothermal wall boundary conditions with non-adiabatic chemistry tabulation in combination with radiation modeling

**Fig. 16:**
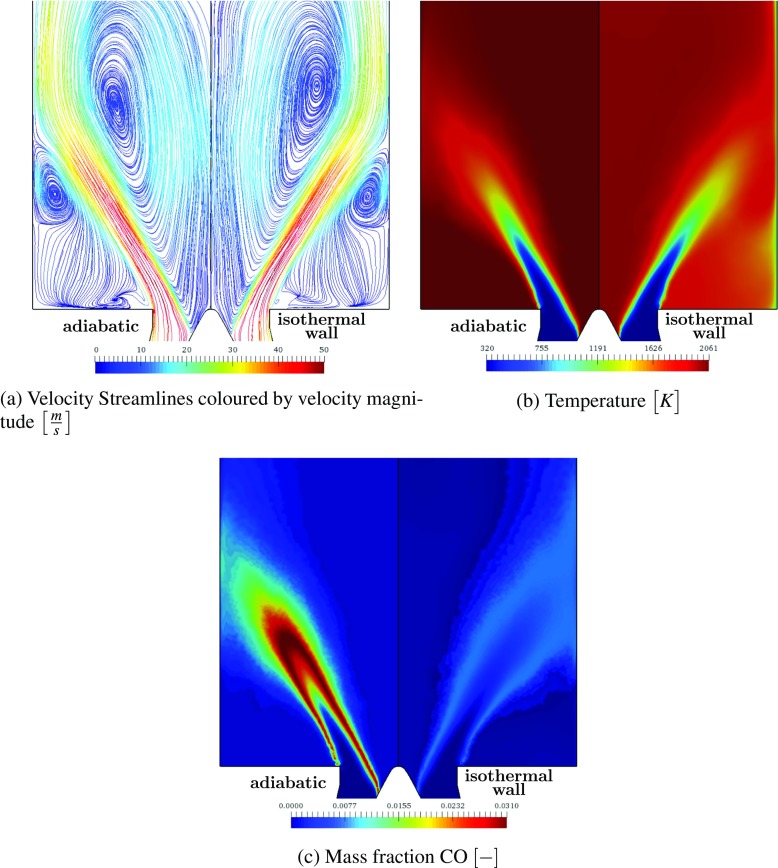
Mean fields for adiabatic (left) and heat loss simulation (right)

In Fig. 16, selected mean fields are compared for the adiabatic (Case 1) and isothermal simulation with the chemical kinetics affected by the heat loss (Case 3). Due to the subdivision of the inlet section upstream of the swirler into three passages, small asymmetries might appear in the solution fields. To assure that this does not affect the comparison, the results corresponding to the same location are mirrored. The comparison of the streamlines presented in Fig. 16a reveals a small impact of the heat loss on the velocity fields. Only the center of the inner recirculation zone is slightly shifted downstream. However, a significant impact of the heat loss on the temperature field is found, so this requires further attention, see Fig. 16b. Due to the isothermal wall condition, the temperature close to the wall and in the ORZ is significantly reduced, which also leads to a lower temperature in the IRZ.

**Fig. 17:**
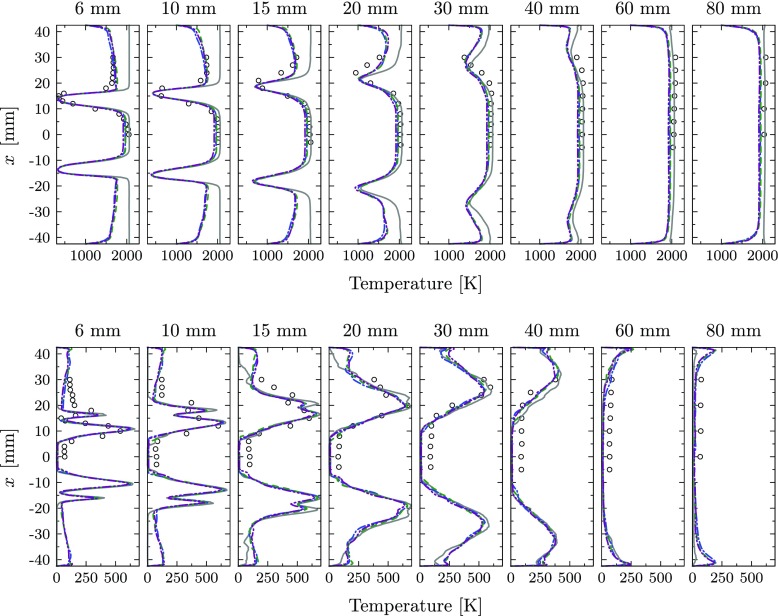
Mean temperature (top) and RMS profiles (bottom): Experiments (), perfectly premixed, adiabatic simulation (), perfectly premixed, non-adiabatic simulation in which the chemistry is not affected by heat loss (), perfectly premixed, non-adiabatic simulation () and perfectly premixed, non-adiabatic simulation including radiative heat transfer ()

In Fig. 17, a quantitative comparison for the profiles of time-averaged and RMS temperature values at the same locations indicated in Fig. 8 is presented. The four cases introduced above are compared with the experimental reference. Significant differences are found between the adiabatic baseline case and the heat loss simulations. The application of isothermal conditions reduces the global temperature in all hot regions of the combustion chamber, while the temperature in the IRZ is only slightly affected. At 20 mm above the burner nozzle, the predicted temperature on the centerline is about 2060 K for Case 1, 1960 K for Case 2, 1970 K for Case 3 and 1915 K for Case 4, while a value of 2030 K was measured in the experiments. However, a major impact is found in regions close to the walls and in the ORZ, where the temperature is strongly reduced for the heat loss simulations. It is evident that the addition of heat loss significantly improves the correlation with the experimental data at all locations. By application of isothermal conditions, a temperature reduction between 300 and 500 K is found in the ORZ. The inclusion of heat loss to the walls also improved the prediction of temperature fluctuations. The RMS peak values are reduced compared to the adiabatic baseline case, but the most significant difference is obtained for the ORZ close to the burner plate. In this region, the RMS value is increased by about 150 K. The results show only a negligible effect of non-adiabatic chemical kinetics on the prediction of the flame dynamics for the current test case. Both, mean and RMS temperature values do only slightly differ for the two non-adiabatic cases with and without heat loss in the chemistry. This can be explained by the compactness of the flame in combination with the high momentum. This results in short residence times in the reacting layer and a small flame surface and therefore low impact of heat loss on the reaction rates. Additionally, the inner reacting layer is hardly affected by the heat loss to the outer walls. The small effect of the heat loss on the chemical kinetics also explains the good predictions on flame dynamics reported in previous studies of this test case, in which adiabatic thermal conditions were systematically applied.

The comparison of Cases 3 and 4 reveals a small effect of radiative heat transfer for this test case. The temperature in regions at equilibrium conditions is reduced about 50 K, but colder regions and flame shape are not significantly affected. This can be explained by the small residence time in the hot flame zone.

**Fig. 18:**
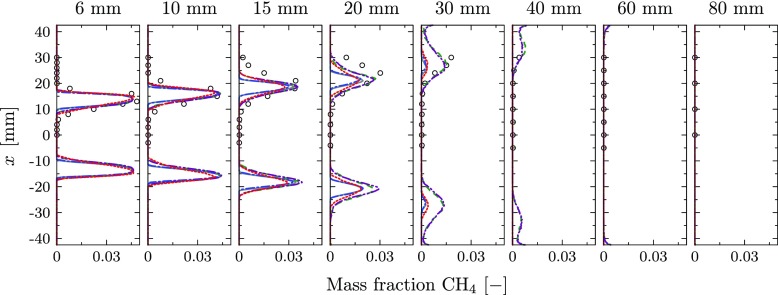
CH_4_ mass fractions. Experiments (), perfectly premixed, adiabatic simulation (), perfectly premixed, non-adiabatic simulation in which the chemistry is not affected by heat loss (), perfectly premixed, non-adiabatic simulation (), perfectly premixed, non-adiabatic simulation including radiative heat transfer ( ) and non-premixed simulation ()

The predicted CO mass fraction visualized in Fig. 16c reveals highly reduced peak values in the reacting layer for the isothermal simulation, indicating that the chemical composition is effected by heat loss. The profiles of CH_4_ mass fraction are presented in Fig. 18 comparing the different simulation results with the experiments. As the mass fractions are directly linked to the controlling variables, the figure is representative for all major species.

In general, the peak locations are well captured by all simulations. However, the strength of the predicted peak value is significantly affected by the influence of the heat loss on the chemical kinetics. While the equilibrium conditions are almost identical for all cases, the prediction of the composition in the reacting layer is significantly improved for the simulations with non-adiabatic chemistry tabulation (Cases 3 and 4).

**Fig. 19:**
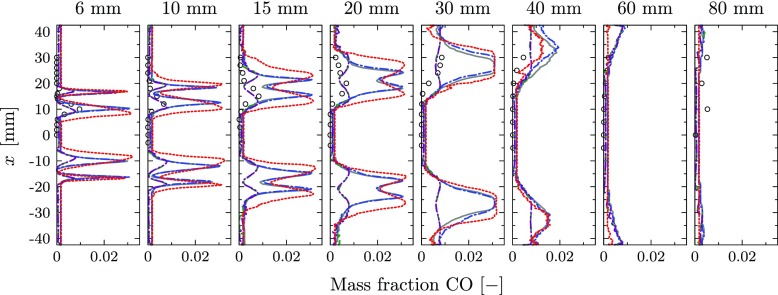
CO Mass fraction. Experiments (), perfectly premixed, adiabatic simulation (), perfectly premixed, non-adiabatic simulation in which the chemistry is not affected by heat loss (), perfectly premixed, non-adiabatic simulation (), perfectly premixed, non-adiabatic simulation including radiative heat transfer () and partially premixed simulation ()

A similar effect is observed for the mass fraction profiles of CO, which are given in Fig. 19 as an example of intermediate species. All adiabatic chemistry simulations predict large peaks in the reacting layers with values of about 0.03. These peak values are reduced in the simulations including the effect of heat loss on the chemistry, leading to maximum values of about 0.0075. This is in alignment with the measured values, even though large measurement uncertainties of up to 50% are reported for CO [4].

The predicted species mass fractions from the non-premixed simulation are also added for completeness. Compared to the baseline case, the prediction of the major species slightly improves, but due to the neglected heat loss, large deviations are found in the reacting layer.

The comparison between the isothermal simulations in which the effect of heat loss on the chemical kinetics is either including or neglecting reveals only negligible differences for the predicted temperature field. However, a clear improvement of the predicted chemical composition is observed for the case in which the chemical kinetics are influenced by the local temperature (Cases 3 and 4).

**Fig. 20:**
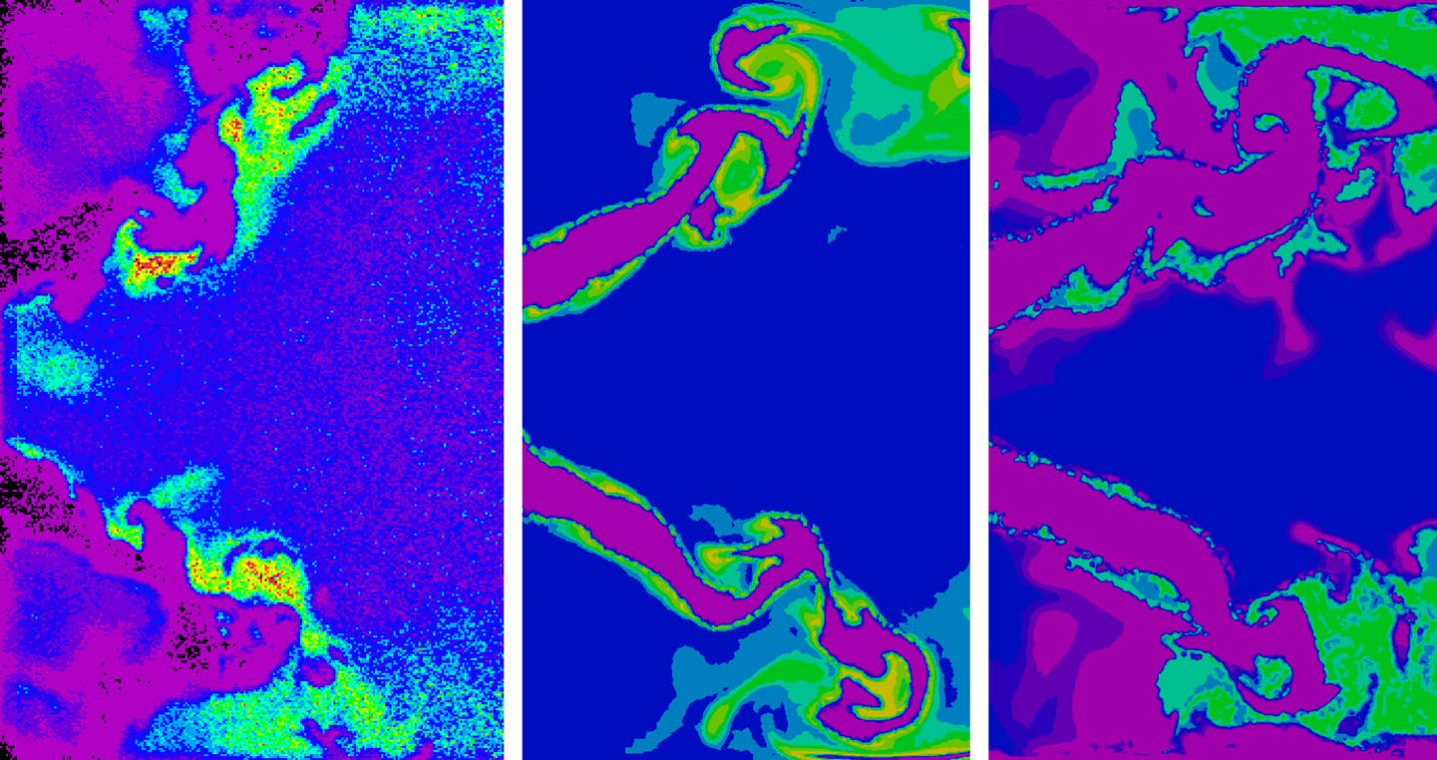
Comparison of OH-LIF measurements [4] (left) with the OH concentrations of the adiabatic simulation (middle) and the non-adiabatic simulation (right)

An important parameter to evaluate the influence of heat loss is the analysis of the OH distribution. In the framework of the measurement campaign at DLR, laser-induced fluorescence (LIF) is used to visualize the OH radical concentration in the combustion chamber [4]. In Fig. 20, the measured OH distribution is compared to the OH concentration predicted by the adiabatic and non-adiabatic LES. Since, the measurements only provide qualitative results, the comparison is mainly intended to provide a global overview of the flame structure. In general, one of the main impacts of the heat loss is the reduction of the OH concentrations, which in this case is about one order of magnitude lower with respect to the adiabatic simulations. Additionally, the gradients in the flame front are also reduced, while the flame thickness is slightly increased. In comparison to the experiments, the OH concentration of the adiabatic simulation is overpredicted in the ORZ and a distinctive M-shaped flame is observed. This is in agreement with the DNS results reported by Moureau et al. [18]. With the existence of heat loss, the flame transitions from the pronounced M-shape to a V-shape manly by the dilution caused by burnt gasses at different enthalpy levels and by the reduction of the OH levels in the ORZ. These results show clearly this transition, even though the OH concentration is still overpredicted in the vicinity of the dump plane compared to the experimental reference. This can be explained by the adiabatic condition prescribed at these walls in the simulation. Nevertheless, also the measurements seem to be slightly disturbed by the impact of the walls in these regions. While the experiments only predict large peak values of OH concentration in the inner shear layer, the simulation additionally predicts a thin region of high values in the outer reacting layer. As the predicted temperature in this region is very much in agreement with the experiments, this difference can be related to the mesh resolution. While the resolution is sufficient to capture the evolution of temperature and major species concentrations, the accurate prediction of small scale intermediate species with short time and small spatial scales (peak values of about 1e-6 are observed for OH concentrations) requires a higher resolution in the reacting layer.

## Conclusions

In this work, the modeling of a swirl stabilized gas turbine model combustor is addressed by means of LES. The combustion chemistry is described by laminar premixed flamelets and presumed shape probability density functions for turbulence chemistry interaction in large-eddy simulation. The prediction capabilities of the combustion model for perfectly premixed and partially premixed conditions are demonstrated and good overall agreement with the experimental data is observed.

In a first section, the influence of non-perfect premixing on the reacting flow field is assessed by comparison of perfectly premixed and partially premixed simulation results. Even though the fuel-air mixture is not homogeneous, many previous investigations are based on the assumption of perfectly premixed conditions. The flame index obtained in the partially premixed simulation also confirms that flame propagation dominates the combustion process in this case, and small influence of diffusion occurs on the postflame region. The analysis revealed significant mixture fraction fluctuations with a scattering between 0.03 and 0.07 and RMS peak values of about 15% of the mean value. However, only small impact of these fluctuations on the turbulent reacting flow field is observed. The velocity profiles reveal a correct prediction of the main flow features for both, perfectly and partially premixed conditions. The time-averaged temperature as well as the RMS values are accurately captured by both simulations apart from the ORZ, where a significant overprediction of the temperature is observed due to neglected heat loss.

In a second section, the influence of heat loss is discussed. Adiabatic thermal conditions are employed in almost all previous modeling attempts of this test case. The neglected heat loss to the walls causes an overprediction of the mean temperature close to the chamber walls, especially in the ORZ. As a consequence, an underprediction of the temperature fluctuations for this region is found. Isothermal boundary conditions are applied for the quartz glass walls of the combustion chamber and significant improvement of the temperature prediction is observed. The time-averaged temperature in the ORZ is reduced between 300 and 500 K, which is in agreement with the measured values. The RMS prediction in this region is increased by about 150 K and improves the alignment with the reference data. The comparison between heat loss simulations using non-adiabatic chemistry tabulation and with a thermo-chemical database in which the effect of heat loss on the chemical kinetics is neglected shows only negligible influence on the prediction of the flame dynamics. This also explains the good prediction of the flame shape in many of the previously reported studies of this combustor, in which adiabatic conditions were assumed. The application of a radiation model based on the optically thin gas assumption does not reveal a major impact of radiative heat transfer for the current case. The equilibrium temperature is reduced by about 50 K, but the flame dynamics are not affected.

Despite small impact is observed on the temperature field and flame dynamics by non-adiabatic simulations with adiabatic kinetics, significant differences are found on the chemical structure of the flame. The application of non-adiabatic chemistry tabulation improves the prediction of the major species in the reacting layers, leading to very good agreement with the measured values. In particular, the prediction of intermediate species like CO is remarkably improved, for which significantly overpredicted values are observed if the heat loss effect on the chemistry is neglected. The comparison of the OH concentrations additionally demonstrates the importance of heat loss modeling to predict the flame shape, which in this case transitions from M- to V-shape by the existence of heat loss.
